# Reduction of Multidrug-Resistant (MDR) Bacterial Infections during the COVID-19 Pandemic: A Retrospective Study

**DOI:** 10.3390/ijerph18031003

**Published:** 2021-01-23

**Authors:** Enrico Bentivegna, Michelangelo Luciani, Luca Arcari, Iolanda Santino, Maurizio Simmaco, Paolo Martelletti

**Affiliations:** 1 Internal Medicine and Emergency Medicine, St’Andrea Hospital, Sapienza University, 00189 Rome, Italy; michelangeloluciani@gmail.com (M.L.); paolo.martelletti@uniroma1.it (P.M.); 2 Covid-Cardiology Unit, Madre Giuseppina Vannini Hospital, 00177 Rome, Italy; luca.arcari88@gmail.com; 3 Hospital Direction and Clinical Departments, St’Andrea Hospital, Department of Neurosciences, Mental Health and Sensory Organs, Sapienza University, 00189 Rome, Italy; iolanda.santino@uniroma1.it (I.S.); maurizio.simmaco@uniroma1.it (M.S.); 4 Department of Clinical and Molecular Medicine, Sapienza University, 00189 Rome, Italy

**Keywords:** COVID-19, SARS-CoV-2, multidrug resistant, prevention, hygiene, PPI, nosocomial infections, hospital-acquired infections

## Abstract

Multidrug-resistant (MDR) organisms are emerging as some of the main healthcare problems worldwide. During the COVID-19 pandemic, several Infection Prevention and Control (IPC) measures have been adopted to reduce nosocomial microorganism transmission. We performed a case–control study to identify if the incidence of MDR bacterial infections while using pandemic-related preventive measures is lower than in previous years. From 2017 to 2020, we monitored hospital discharges over a four-month period (P #) (1 March to 30 June) in St. Andrea Hospital, Rome. In total, we reported 1617 discharges. Pearson’s chi-squared test was used to identify significant differences. A value of *p* ≤ 0.05 was considered statistically significant. A significant reduction in the incidence of total MDR bacterial infections was observed during the pandemic compared to in prepandemic years (*p* < 0.05). We also found a significantly higher incidence of MDR bacterial infections in COVID-19 departments compared with other medical departments (29% and 19%, respectively), with extended-spectrum *β*-lactamase *Klebsiella pneumoniae* as the pathogens presenting the highest increase. This study demonstrates that maintaining a high level of preventive measures could help tackle an important health problem such as that of the spread of MDR bacteria.

## 1. Introduction

Hospital-associated infections (HAIs) are emerging as some of the main healthcare problems worldwide. Recently, there has been increasing attention on this problem and on how to prevent it. Mostly being linked to multidrug-resistant (MDR) organisms, HAIs cause about 99,000 deaths each year in the US [1], with an estimated cost ranging from $4.5 billion to $11 billion per year [2].

The continuous selective pressure dictated by the widespread and sometimes unjustified use of broad-spectrum antibiotics has led to the selection of bacteria increasingly resistant to most modern forms of antibiotics. The race to develop new classes of antibiotics is often counterbalanced by the equally rapid evolution of resistant forms of bacteria.

Since there are few therapeutic options in the case of an infection by extensively resistant or panresistant bacteria, the World Health Organization (WHO) highlights how preventive measures are the most efficient strategy to face the rise of MDR organisms [3]. 

It has been shown how microorganism contamination of healthcare workers’ hands contributes to the spread of HAIs in hospital settings [4], and errors in hygiene procedures are at the base of self-contamination [5]. Despite this evidence, it is still not perfectly clear which preventive procedures are best to effectively reduce the incidence of these hospital complications. Most of them derive from observational studies in contexts in which changes in the way of working within a hospital have been adopted.

Severe acute respiratory syndrome coronavirus-2 (SARS-CoV-2) is a human coronavirus that causes COVID-19, a highly infectious respiratory disease. From Wuhan, China, where the first cases were reported, it rapidly spread to the rest of the world, causing a pandemic [6]. To date, it has caused more than 1,690,000 deaths [7], showing a higher prevalence in regions with high levels of pollution [8]. Similar to MDR bacterial infections, the importance of prevention as the main strategy to eliminate COVID-19 has been emphasized. Due to its high diffusibility and contagiousness, it has triggered a great effort by the scientific community to study and identify important information to counteract its spread. 

During the COVID-19 pandemic, several Infection Prevention and Control (IPC) measures have been adopted to reduce nosocomial microorganism transmission [9,10,11].

A great effort is being made by healthcare personnel to contain the in-hospital spread of the virus. For the first time, the prolonged use of intensive preventive measures, previously adopted only in high-risk units, has been rolled out continuously and on a large scale. Since prevention appears to be the best way to deal with the spread of MDR bacteria and HAIs, great effort has been made by the scientific community to understand the most effective preventive strategies. The overall objective of this study is to provide an overview of MDR bacterial infections over the past four years. 

Analyzing the trend of MDR infections, this study aims to better understand the impact and efficacy of preventive measurements in reducing the incidence of other HAIs. The purpose is to assess whether hygiene measures adopted during the pandemic have a protective role against the spread of MDR bacteria in hospital settings.

## 2. Methods

We performed a case–control study to identify if the incidence of MDR bacterial infections while using pandemic-related preventive measures is lower than in previous years. From 2017 to 2020, we monitored hospital discharges over a 4-month period (P #) (1 March to 30 June) in St. Andrea Hospital, Rome. In total, we reported 1617 discharges.

In our center, before the pandemic, only basic prevention measures to avoid the spread of microorganisms were routinely adopted. Patients infected with MDR bacteria were isolated in the cohort areas, managed with gloves and disposable gowns, and subjected to active surveillance by our hygiene and prevention department. Surgical masks were used only in high-risk units or when dealing with severe neutropenic patients. 

During the pandemic, several additional measures have been adopted: constant use of adequate personal protective equipment, change of gloves and gowns in-between patients, increased attention to hand hygiene, segregation of patients with fever or respiratory symptoms, visiting limitations, universal masking policies, and social distancing measures recommended by the World Health Organization (WHO) [9,10,11].

According to the European Centre for Disease Prevention and Control (ECDC) and the Centers for Disease Control and Prevention (CDC), MDR is defined by a resistance to at least one agent in three or more antimicrobial categories [12]. Diagnosis of *Clostridium difficile* infection is confirmed by the presence of diarrhea plus stool tests positive for *Clostridium difficile*.

MDR bacterial infections that arose during the hospitalization period are reported in Table 1. The rates of MDR infections were calculated as the total number of cases divided by the total number of discharges over the study period. Pearson’s chi-squared test was used to identify significant differences. A value of *p* ≤ 0.05 was considered statistically significant.

We compared the rates of total MDR infections after the introduction of preventive measures with the rate of MDRI over P # 2017, P # 2018, and P #2019. For the four most common bacteria, we individually compared the infection rate during P # 2020 with the average infection rate between P # 2017 and P # 2019. (* = *p* < 0.05).

## 3. Results

We examined 1617 discharges over a four-month period (P #) (1 March to 30 June) from 2017 to 2020 (Table 1). 

**Figure 1:**
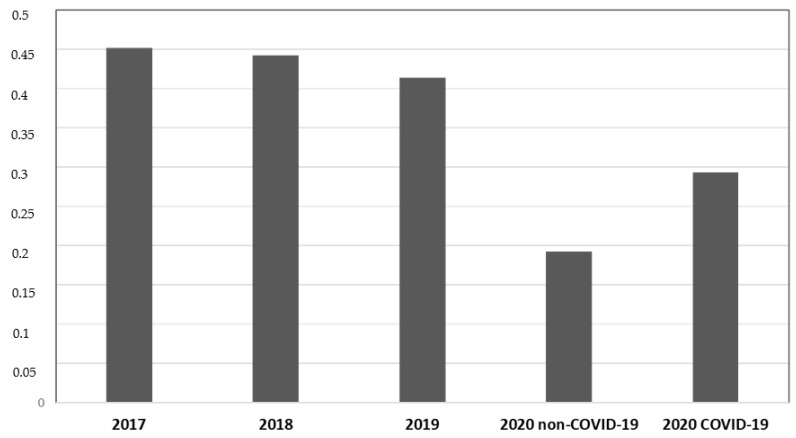
Data from medical departments between 1 March and 30 June. The ordinate axis represents the infection incidence of MDR bacteria. The abscissas axis represents time.

During P # 2020, both COVID-19 (19.2%) and non-COVID-19 (29.2%) departments showed a lower incidence of total MDR bacterial infection compared to previous years (45.2% during P # 2017, 44.2% during P # 2018, and 41.4% during P# 2019) (*p* < 0.05, CIs 95%). COVID-19 departments showed a higher MDR infection incidence than non-COVID-19 departments of the same year (29.2% vs. 19.2%) (*p* < 0.05, CIs 95%). No difference in MDRBI incidence was found between P # 2017, P # 2018, and P # 2019 (Figure 1).

The incidence of total infection by MDR bacteria was 45.2 cases per 100 discharges during P # 2017, 44.2 during P # 2018, 41.4 during P # 2019, 19.2 during P # 2020 in non-COVID-19 departments, and 29.3 during P # 2020 in COVID-19 departments. A significant reduction in the incidence of total MDR bacterial infections was observed during the pandemic compared to in prepandemic years (*p* < 0.05). 

The most common MDR pathogens isolated during the period were in order of frequency: methicillin-resistant *Staphylococcus Aureus*, extended-spectrum *β*-lactamase *Klebsiella pneumoniae*, health care-associated *Clostridium difficile* (HA-CD), and *Acinetobacter baumannii*.

From the analysis of the infection rates of the four most common pathogens, several points deserve to be mentioned. 

During P # 2020, non-COVID-19 departments showed a lower incidence of the four most frequent MDR bacteria compared to previous years (*p* < 0.01). COVID-19 departments showed a higher methicillin-resistant *Staphylococcus aureus*, *Clostridium difficile*, and *Acinetobacter baumannii* infection incidence than non-COVID-19 departments of the same year (not significant). COVID-19 departments showed a significantly higher *Klebsiella pneumoniae* infection incidence than non-COVID-19 departments of the same year (*p* < 0.05).

Methicillin-resistant *Staphylococcus aureus* showed the highest prepandemic incidence and the highest reduction in IPC measures adopted in P # 2020 (from 14 to 4.2 cases per 100 discharges). The difference was statistically significant (*p* < 0.05). 

The mean incidence of extended-spectrum *β*-lactamase *Klebsiella pneumoniae* infection between P # 2017 and P # 2019 was 9.4 cases per 100 discharges. During the pandemic, a statistically significant reduction of its infection incidence in non-COVID-19 departments was observed (4.8 cases per 100 discharges) (*p* < 0.05), but no difference was observed in COVID-19 departments (10.6 cases per 100 discharges). 

**Figure 2:**
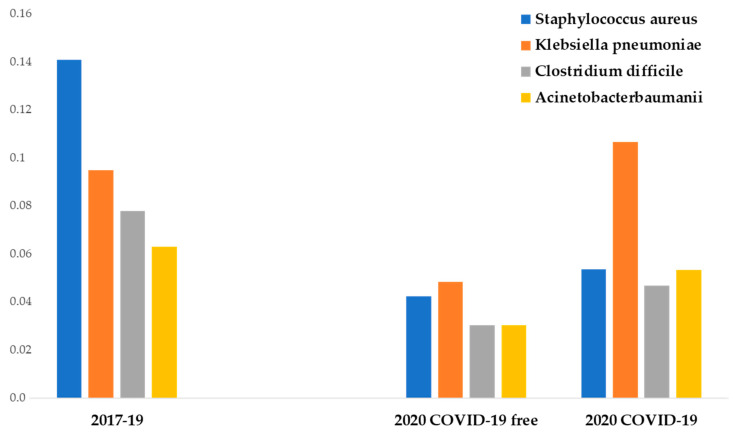
Data from medical departments between 1 March and 30 June. The ordinate axis represents the infection incidence of MDR bacteria. The abscissas axis represents time.

The incidence of *Acinetobacter baumannii* infection between P # 2017 and P # 2019 was 6.3 cases per 100 discharges. During the pandemic, the infection incidence decreased at 3 cases per 100 discharges in non-COVID-19 departments (significant reduction, *p* < 0.05). In a similar way, during P # 2020, a significant reduction of *Clostridium difficile* infection incidence was observed (7.8 vs. 3 cases per 100 discharges) (*p* < 0.05) (Figure 2).

Additional findings emerged comparing COVID-19 departments with other medical departments during the pandemic. In fact, during this period, COVID-19 patients showed a significantly higher incidence of total MDR infections compared with no-COVID-19 patients (19.2 vs. 29.3 cases per 100 discharges) (*p* < 0.05), with extended-spectrum *β*-lactamase *Klebsiella pneumoniae* infection showing the highest increase (4.8 vs. 10.6 cases per 100 discharges) (*p* < 0.05).

## 4. Discussion

The main finding of the study was that the large-scale use of COVID-19 preventive measures led to a significant reduction of all MDR bacteria, principal etiological agents of HAIs.

Although healthcare workers, hygiene education, personal protective equipment (PPE) employments, and contact precautions are strongly recommended, evidence for their efficacy in HAI prevention is still weak. Despite the limited period of observation, our study demonstrates that such strategies are effective in reducing the spread of HAIs and MDR bacteria. 

At the beginning of the pandemic, PPE-like disposable medical coats, latex gloves, and surgical masks were employed by health care personnel as well as the disinfection of surfaces and frequent hand washing. 

Regarding the no-COVID-19 departments, relatives’ visits were reduced from two times to one time per day and were limited to only one family member at a time. Each visitor had to adopt universal masking policies and social distancing measures recommended by the World Health Organization (WHO) [9,10,11]. Any visit from patients’ relatives was totally prohibited in the COVID-19 wards.

Unlike what was observed during the severe acute respiratory syndrome (SARS) outbreak in 2003 [13], when preventive measures led to an increase of methicillin-resistant *Staphylococcus aureus* diffusion, during the COVID-19 pandemic, the incidence of this infection has shown a strong reduction. A study from Lei et al. [14] reported similar results.

Regarding *Clostridium difficile*, as already reported in previous works [15,16], IPC measures adopted during the pandemic have led to a significant reduction of its infection incidence. Conversely, other studies from different institutions have revealed no reduction of *Clostridium difficile* infection incidence during the pandemic [14]. A possible explanation for this discrepancy is that hygiene measures adopted at the institution from Lei et al. were already at high levels before the pandemic, and the IPC measures adopted in 2020 did not have a significant impact.

Another important finding of this study is that COVID-19 patients have a higher incidence of MDR bacterial infections compared to non-COVID-19 patients, despite the fact that IPC measures were even stricter in the first group. This finding could be explained by the widespread use of broad-spectrum antibiotics in COVID-19 patients, which may be responsible for an increase in the incidence and the selection of multidrug-resistant bacteria [17]. Furthermore, COVID-19 departments are mostly managed by infectious disease specialists, who are more likely to perform culture tests. Another possible explanation may be given by the intrinsic characteristics of the patients themselves. In fact, hospitalized COVID-19 patients are often highly comorbid subjects who went through several hospitalizations during previous months. This could have led to colonization by MDRB, which, during the coronavirus disease, was able to produce a full-blown infection. Last but not least, an impaired immune system, characteristic of patients developing severe forms of COVID-19 [18,19,20,21], may have been an important risk factor for MDR bacterial infections. Regarding increased susceptibility to *Clostridium difficile* infections, an alteration of the intestinal microbiota in COVID-19 patients has already been proposed as a possible contributing cause of these HAIs [15].

This study certainly has some limitations. First of all, it was conducted in a single center; therefore, it was difficult to generalize the findings that emerged since MDRB prevalence is often specific to each hospital. Furthermore, our study does not explain which of the measures adopted during emergency played a major role in the reduction of HAIs and MDR bacterial infections. 

Other articles have demonstrated that intensification in hand hygiene practices is not effective in preventing *Clostridium difficile* infections [22]. This discrepancy may be due to the products used in hand washing or by the fact that all the preventive measures used in their entirety have effective action. 

Another limitation of this study is that it does not take into account the consumption of antibiotics during the periods analyzed. It is well known that the incidence of MDR bacteria is directly proportional to the excessive and inappropriate use of antibiotics, which select resistant strains of bacteria. As previously described, one of the explanations for the higher incidence of bacteria in COVID-19 patients is that often in these departments, there is broad-spectrum antibiotic use.

However, the patients compared in the non-COVID-19 wards from P # 2017 to P # 2020 were all from the medical departments of the same structure; thus, it is assumed that, on average, there has been the same use of antibiotics over the years.

We can speculate that greater attention paid by health personnel along with all preventive measures in their complex has led to the reduction in MDR bacterial infections. A possible take-home message (recommending the use of IPC measures even after the pandemic) would clash with several logistical and organizational problems, requiring a radical change in the working methods of healthcare personnel.

## 5. Conclusions

This study demonstrates that maintaining a high level of preventive measures could help tackle an important health problem such as the spread of HAIs and MDRB. The COVID-19 pandemic and its associated healthcare efforts have given us the opportunity to better understand effective measures for HAI and MDRB prevention. These findings do not reveal which of the measures adopted during the emergency played a major role in HAI reduction. We can speculate that all practices adopted in their complex led to a smaller spread of MDR organisms. Herein, we also identify the pathogen most sensitive to such measures and the most common infectious agents in COVID-19 patients.

We hope this study will foster a better understanding of the risk factors related to MDR bacterial infections and promote the use of effective preventive measures even after the pandemic.

## Figures and Tables

**Table 1:** Data from medical departments during the period (P#) from 1 March to 30 June in 2017, 2018, 2019 and 2020. The first row indicates the year analyzed. During P# 2020, COVID-19 and non-COVID-19 departments were analyzed separately. The second row shows the number of discharges. The following rows show the infection incidence of each pathogen. The last row indicates the total incidence of infections from any type of multidrug-resistant (MDR) bacteria. The *p*-value refers to the years before the pandemic.

Year	P # 2017	P # 2018	P # 2019	P # 2020 Non-COVID-19	P # 2020 COVID-19
Discharges	422	348	364	333	150
*Staphylococcus aureus*	7.7%	13.8%	15.4%	4.2% *	5.3%
*Klebsiella pneumoniae*	12%	6.6%	9%	4.8% *	10.6%
*Clostridium difficile*	9.2%	7.2%	6.6%	3.3% *	4.7%
*Acinetobacter baumannii*	6.1%	8%	4.7%	3% *	5.3%
*Escherichia coli*	0.4%	0.6%	0.3%	0.3%	0%
*Enterococcus faecium*	2.4%	2.6%	2.7%	2.7%	2%
*Pseudomonas aeruginosa*	1.2%	3.4%	2.2%	0.6%	1.3%
*Enterococcus Faecalis*	0.7%	1.4%	0.3%	0.3%	0%
*Stenotrophomonas maltophilia*	0%	0.6%	0%	0%	0%
*Klebsiella variicola*	0%	0%	0.3%	0%	0%
Total	45.2%	44.2%	41.4%	19.2% *	29.2%

## Data Availability

The data presented in this study are available on request from the corresponding author. The data are not publicly available due to privacy restrictions.

## References

[1] Klevens R.M., Edwards J.R., Richards C.L., Horan T.C., Gaynes R.P., Pollock D.A., Cardo D.M. (2007). Estimating health care-associated infections and deaths in U.S. hospitals, 2002. Public Health Rep..

[2] Hospital-Acquired Infection (HAI) Diagnostics Market is Forecasted to Reach $4,386.6 Million by 2023, Growing at a CAGR of 7.6% During 2017–2023, P&S Intelligence. https://www.psmarketresearch.com/market-analysis/hospital-acquired-infection-diagnostics-market.

[3] Chan M. Antimicrobial Resistance. https://www.who.int/news-room/fact-sheets/detail/antimicrobial-resistance.

[4] Pratt R.J., Pellowe C., Loveday H.P., Robinson N., Smith G.W., Barrett S., Davey P., Harper P., Loveday C., McDougall C. (2001). Department of Health (England). The epic project: Developing national evidence-based guidelines for preventing healthcare associated infections. Phase I: Guidelines for preventing hospital-acquired infections. Department of Health (England). J. Hosp. Infect..

[5] Kwon J.H., Burnham C.D., Reske K.A., Liang S.Y., Hink T., Wallace M.A., Shupe A., Seiler S., Cass C., Fraser V.J. (2017). Assessment of healthcare worker protocol deviations and self-contamination during personal protective equipment donning and doffing. Infect. Control. Hosp. Epidemiol..

[6] Takagi G., Yagishita K. (2020). Principles of disinfectant use and safety operation in medical facilities during coronavirus disease 2019 (COVID-19) outbreak. SN Compr. Clin. Med..

[7] Zhu N., Zhang D., Wang W., Li X., Yang B., Song J., Zhao X., Huang B., Shi W., Lu R. (2020). A novel Coronavirus from patients with *Pneumonia* in China, 2019. China novel Coronavirus investigating and research team. N. Engl. J. Med..

[8] https://github.com/CSSEGISandData/COVID-19.

[9] Martelletti L., Martelletti P. (2020). Air pollution and the novel Covid-19 disease: A putative disease risk factor. SN Compr. Clin. Med..

[10] Wee L.E., Sim X.Y.J., Conceicao E.P., Aung M.K., Goh J.Q., Yeo D.W.T., Gan W.H., Chua Y.Y., Wijaya L., Tan T.T. (2020). Containment of COVID-19 cases among healthcare workers: The role of surveillance, early detection, and outbreak management. Infect. Control. Hosp. Epidemiol..

[11] Wee L.E., Hsieh J.Y.C., Phua G.C., Tan Y., Conceicao E.P., Wijaya L., Tan T.T., Tan B.H. (2020). Respiratory surveillance wards as a strategy to reduce nosocomial transmission of COVID-19 through early detection: The experience of a tertiary-care hospital in Singapore. Infect. Control. Hosp. Epidemiol..

[12] Magiorakos A.P., Srinivasan A., Carey R.B., Carmeli Y., Falagas M.E., Giske C.G., Harbarth S., Hindler J.F., Kahlmeter G., Olsson-Liljequist B. (2012). Multidrug-resistant, extensively drug-resistant and pandrug-resistant bacteria: An international expert proposal for interim standard definitions for acquired resistance. Clin. Microbiol. Infect..

[13] Yap F.H., Gomersall C.D., Fung K.S., Ho P.L., Ho O.M., Lam P.K., Lam D.T., Lyon D.J., Joynt G.M. (2004). Increase in methicillin-resistant Staphylococcus aureus acquisition rate and change in pathogen pattern associated with an outbreak of severe acute respiratory syndrome. Clin. Infect. Dis..

[14] Wee L.E.I., Conceicao E.P., Tan J.Y., Magesparan K.D., Amin I.B.M., Ismail B.B.S., Toh H.X., Jin P., Zhang J., Wee E.G.L. (2020). Unintended consequences of infection prevention and control measures during COVID-19 pandemic. Am. J. Infect. Control.

[15] Bentivegna E., Alessio G., Spuntarelli V., Luciani M., Santino I., Simmaco M., Martelletti P. (2020). Impact of COVID-19 prevention measures on risk of health care-associated Clostridium difficile infection. Am. J. Infect. Control.

[16] Ponce-Alonso M., Sáez de la Fuente J., Rincón-Carlavilla A., Moreno-Nunez P., Martínez-García L., Escudero-Sánchez R., Pintor R., García-Fernández S., Cobo J. (2020). Impact of the coronavirus disease 2019 (COVID-19) pandemic on nosocomial *Clostridioides difficile* infection. Infect. Control Hosp. Epidemiol..

[17] Liew Y., Lee W.H.L., Tan L., Kwa A.L.H., Thien S.Y., Cherng B.P.Z., Chung S.J. (2020). Antimicrobial stewardship programme: A vital resource for hospitals during the global outbreak of coronavirus disease 2019 (COVID-19). Int. J. Antimicrob. Agents.

[18] Luciani M., Bentivegna E., Spuntarelli V., Amoriello Lamberti P., Guerritore L., Chiappino D., Nalli G., Proietta M., Del Porto F., Martelletti P. (2020). Coinfection of tuberculosis pneumonia and COVID-19 in a patient vaccinated with Bacille Calmette-Guérin (BCG): Case report. SN Compr. Clin. Med..

[19] Bentivegna E., Luciani M., Spuntarelli V., Speranza M.L., Guerritore L., Sentimentale A., Martelletti P. (2020). Extremely severe case of COVID-19 *Pneumonia* recovered despite bad prognostic indicators: A didactic report. SN Compr. Clin. Med..

[20] Bentivegna E., Sentimentale A., Luciani M., Speranza M.L., Guerritore L., Martelletti P. (2020). New IgM seroconversion and positive RT-PCR test after exposure to the virus in recovered COVID-19 patient. J. Med. Virol..

[21] Luciani M., Bentivegna E., Spuntarelli V., Lamberti P.A., Cacioli G., del Porto F., Sesti G., Martelletti P., de Biase L. (2020). Recurrent COVID-19 pneumonia in the course of chemotherapy: Consequence of a weakened immune system?. J. Med. Virol..

[22] Louh I.K., Greendyke W.G., Hermann E.A., Davidson K.W., Falzon L., Vawdrey D.K., Ting H.H. (2017). Clostridium difficile infection in acute care hospitals: Systematic review and best practices for prevention. Infect. Control Hosp. Epidemiol..

